# 2321: Soluble adenylyl cyclase (sAC) regulates melanogenesis and melanocyte response to UVB

**DOI:** 10.1017/cts.2017.40

**Published:** 2018-05-10

**Authors:** Koji Ota, Dalee Zhou, Jonathan Zippin

## Abstract

OBJECTIVES/SPECIFIC AIMS: Our objective is to study the role of soluble adenylyl cyclase in the melanocyte regulation of pigment in response to ultraviolet radiation. Melanocytes are specialized cells that produce melanin in organelles called melanosomes, and melanin determines the pigmentation of hair and skin. cAMP is a master regulator of pigmentation and transmembrane class of adenylyl cyclases are essential for expression of important enzymes involved in melanogenesis. However, pigmentation is also controlled by melanosomal pH, which regulates melanogenesis, tyrosinase activity, and melanosome maturation. The relationship between melanosomal pH and cAMP has been elusive. Soluble adenylyl cyclase is a noncanonical source of cAMP that is not responsive to G proteins but rather functions as a pH sensor. We recently demonstrated that loss of soluble adenylyl cyclase (sAC) activity leads to increased melanosomal pH as well as increased pigmentation in cells and hair. We expanded our research to investigate the role of sAC in the intrinsic response of melanocytes to ultraviolet radiation. METHODS/STUDY POPULATION: We utilized sACfl/fl (wild type) and sACKO mouse melanocytes and compared their change in pigmentation in response to ultraviolet radiation. Melanin was used as a measure of pigmentation. We irradiated these cells at differing doses of UVB (0, 1, 2, or 3 mJ/cm^2^) daily for 3 days. After UVB treatment, cells were observed and the surviving cell numbers were determined. Cells were then analyzed for melanin content using spectroscopy. RESULTS/ANTICIPATED RESULTS: We found that while both sACfl/fl and sACKO cells had increased melanin content in response to UVB, the melanin content of sACKO cells increased more compared with sACfl/fl cells (*p*=0.001 at daily dose of 3 mJ/cm^2^). In addition, sACKO cells required less UVB dose to induce a response. We also observed that sACKO cells show increased cell death compared with sACfl/fl cells. DISCUSSION/SIGNIFICANCE OF IMPACT: Although both sACfl/fl and sACKO cells can induce melanin production in response to UV, our results suggest that sACKO cells are more sensitive. We believe that this increased response in sACKO cells is due to increased melanosomal pH. In addition, sACKO cells show increased cell death, suggesting that sAC is important in the damage response secondary to UV exposure. UV plays a wide range of roles in skin biology such as contributing to cancer risk and pigmentation. Since pigmentation is essential for the protection of the skin from UV insult, further investigation of possible mechanisms in which sAC can influence pigmentation in response to UV is warranted.

